# Structural Characterization and Physical Properties of Double Perovskite La_2_FeReO_6+δ_ Powders

**DOI:** 10.3390/nano12020244

**Published:** 2022-01-13

**Authors:** Qingkai Tang, Xinhua Zhu

**Affiliations:** National Laboratory of Solid State Microstructures, School of Physics, Nanjing University, Nanjing 210093, China; qktang@mail.nwpu.edu.cn

**Keywords:** double perovskite La_2_FeReO_6+__δ_ powders, solid-state reaction, dielectric properties, magnetic properties, optical properties, structural characterization

## Abstract

The structural, optical, dielectric, and magnetic properties of double perovskite La_2_FeReO_6+__δ_ (LFRO) powders synthesized by solid-state reaction method under CO reduced atmosphere are reported on in this paper. Reitveld refinements on the XRD data revealed that the LFRO powders crystallized in an orthogonal structure (*Pbnm* space group) with column-like morphology. The molar ratios of La, Fe, and Re elements were close to 2:1:1. XPS spectra verified the mixed chemical states of Fe and Re ions, and two oxygen species in the LFRO powders. The LFRO ceramics exhibited a relaxor-like dielectric behavior, and the associated activation energy was 0.05 eV. Possible origins of the dielectric relaxation behavior are discussed based on the hopping of electrons among the hetero-valence ions at B-site, oxygen ion hopping through the vacant oxygen sites, and the jumping of electrons trapped in the shallower level created by oxygen vacancy. The LFRO powders display room temperature ferromagnetism with Curie temperature of 746 K. A Griffiths-like phase was observed in the LFRO powders with a Griffiths temperature of 758 K. The direct optical band gap of the LFRO powders was 2.30 eV, deduced from their absorption spectra, as confirmed by their green photoluminescence spectra with a strong peak around 556 nm.

## 1. Introduction

With the development of modern microelectronics, transistor manufacturing technology based on electronic charge transportation faces great challenges—such as the continuous reduction in the feature size and the size effect, increased leakage current, and so on—which stimulate the development of spintronics and spintronic devices that are based on the electron spin and the spin half-metallic (HM) materials (also called HM materials) [1,2]. HM materials are defined as the materials in which only one-spin direction is present at the Fermi level. Therefore, the density of states of HM materials is fully spin-polarized at the Fermi level. Therefore, HM materials have promising applications in the fields of spintronic devices [3,4,5]. The concept of HM was first proposed in 1983 by de Groot et al. during their theoretical calculations of the band structures of magnetic semi-Heusler compounds [6]. Since then, much effort has been devoted to HM materials used for spin electronics devices [7,8,9]. For examples, several candidates have been discovered to exhibit HM properties, which include (i) semi-Heusler [6] and full-Heusler alloys [10]; (ii) rutile structured oxides such as CrO_2_ [11]; (iii) spinel structured compounds such as magnetite Fe_3_O_4_ [12]; (iv) perovskite structured oxides such as La_0.7_Sr_0.3_MnO_3_ [13]; (v) double perovskite (DP) oxides such as Sr_2_FeMoO_6_ [14] and Sr_2_FeReO_6_ [15]; (vi) zinc blended structured materials such as CrAs, CrTe, and CrSe [16]; (vii) dilute magnetic semiconductors (e.g., (GA, Mn)As, Li_1+x_(Zn, Mn)As) [17]; and (viii) organic half-metal ferromagnets [18]. Among them, ferromagnetic (FM) DP oxides are the most promising candidate for the realization and development of the HM concept because of their simple crystal structure, wide range of magnetic properties, high Curie temperature (*T*_C_), as well as large spin polarization [19,20]. 

To date, numerous DP oxides have been widely studied to search for possible high-*T*_C_ HM materials [21,22,23]. It is found that ordered DP oxides display high *T*_C_ ferrimagnetism and half-metallicity, which are considered to be new kinds of HM materials [24,25]. Among the family of ordered DP oxides, rhenium (Re)-based DP oxides have considerable interest due to their half-metallicity and ferrimagnetism with *T*_C_ much over room temperature as well as large low-field magnetoresistance (coming from the intergrain tunneling effect) [26,27]. In addition, the combination of 3d and 5d transition metal ions at B-site of the re-based DP compounds exhibit abundant electronic structures and complex magnetic structures. That is ascribed to the strong interactions between the 3d and 5d electrons, where the 3d electrons are strongly localized whereas the 5d electrons are highly delocalized with a strong spin-orbital coupling. Recently, Re-based DP oxides such as A_2_MReO_6_ (A_2_ = Ca_2_, Sr_2_, BaSr and Ba_2_; M = Mn, Cr, Fe, Co, Ni) have been widely investigated, which exhibit high *T*_C_ values (e.g., 520 K for Ca_2_CrReO_6_, 410 K for Sr_2_CrReO_6_, 305 K for Ba_2_CrReO_6_, and 610 K for Sr_2_CrReO_6_) and HM properties [28,29]. Expansion studies on HM materials within the Sr-based DP oxides of Sr_2_B′B″O_6_ (where B′ = Co, Cu, and Ni; B″ = Mo, W, Tc, and Re; and B′B″ = FeTc) have been carried out by first-principle theoretical calculations [30]. Recently, the possible candidates of HM-antiferromagnets (HM-AFMs, its total magnetic moment is zero) in LaAB′B″O_6_ DP oxides have been thoroughly investigated based on first-principle calculations [31]. It is predicted that LaAWB″O_6_ DP oxides (where A = Ca, Sr and Ba; B″ = Tc, Re) are the promising candidates of HM-AFMs, where LaAWReO_6_ (A = Sr, Ba) are the better ones under the structural optimization. Analogously, La_2_VReO_6_ DP oxides are also proven to be good candidate for HM-AFM [32]. In the meantime, first-principle theoretical calculations are also carried out to simulate the structural evolution and bonding pattern formation of the compound systems that exhibit a similar level of complexity (e.g., structural and bonding patterns/disorder are dependent upon the precursors during magnetron sputtered deposition of CFx films in CF_4_/Ar atmosphere) [33,34,35]. In recent years, magnetic semiconductors or insulators have also attracted great attention due to their possible applications in spintronic devices [36]. To find the new FM semiconductors with high *T*_C_ from DP oxides, the physical properties of the La_2_B′B″O_6_ (B′B″ pairs = any pair taken from the 29 transition metal elements except La) DP oxides are theoretically calculated [37]. The results show that the ordered La_2_FeB″O_6_ (B″ = Co, Rh, and Ir) DP oxides are FM semiconductor materials. As one of the Re-based DP oxides, La_2_FeReO_6_ (LFRO) has an orthorhombic crystal structure with *Pbnm* space group, exhibiting room temperature magnetism with *T*_C_ of 729 K [38]. A Griffiths phase was also reported in this compound with Griffiths temperature (*T*_G_) of 863 K. It is expected that the LFRO compound could exhibit HM behavior since the strong electron–electron interactions in this DP oxide contribute to their half-metallicity [7]. However, to date, the structural and physical properties of the LFRO DP oxides have not been fully investigated, which are important to their potential applications in spintronics. In the present work, La_2_FeReO_6+δ_ DP oxides are prepared by solid-state reaction method under CO reducing atmosphere. Their structural, dielectric, magnetic, and optical properties are comprehensively investigated. 

## 2. Materials and Methods

### 2.1. Raw Materials

Lanthanum(Ⅲ) oxide (La_2_O_3_, 99.99%), ferric oxide (Fe_2_O_3_, 99.9%), and activated carbon (C, Medical grade) were purchased from Aladdin Reagent limited liability company (Shanghai, China). Rhenium powder (Re ≥ 99.9%) were obtained from Sigma-Aldrich Reagent Ltd. (Shanghai, China). All the chemicals were utilized as received without further purification.

### 2.2. Synthesis of LFRO Powder

The La_2_FeReO_6_ precursors were prepared by solid-state reaction method. First, La_2_O_3_ (325.81 mg, 1 mmol), Fe_2_O_3_ (79.84 mg, 0.5 mmol), and Re (186.21 mg, 1 mmol) were weighted and mixed in a mortar, and then milled in a crucible for 30 min by hands. The milled powders were annealed at 800 °C for 12 h in a muffle furnace. The obtained product was ground again for 30 min, and then sintered at 1000 °C for 12 h in air atmosphere.

The LFRO precursor and active carbon (0.1 g) were mixed in a mortar, and the mixture was ground for 20 min. Then the mixture was pelletized under a cold isostatic pressure and annealed at 1100 °C for 3 h in a closed chamber to obtain the LFRO ceramic samples. The LFRO powders were obtained from the ground ceramic pellets, which were used for the structural characterizations and physical property measurements.

### 2.3. Sample Characterization

X-ray diffraction (XRD) patterns of the LFRO precursors and LFRO powders were collected by using Cu K_α_ radiation and an X-ray diffractometer (Bruker D8 Advance, Bruker AXS GmbH, Karlsruhe, Germany). The scanning angles varied from 2*θ* = 10° to 80° with a step size of 0.02°. The structural refinements of the LFRO powders were performed by the Rietveled method using the GSAS software (http://doc.wendoc.comnl.gov/public/gsas/, accessed on 25 January 2007, GSAS-II, Los Alamos National Laboratory) [39]. Microstructures of the LFRO powders from the ground ceramic pellets were investigated by field-emission scanning electron microscopy (FE-SEM, Zeiss, Sigma 300, Jena, Germany) as well as energy dispersive X-ray spectroscopy (EDS, EX-250 spectroscopy, HORIBA Corporation, Kyoto, Japan). The oxidation states of the La, Fe, and Re and O elements in the LFRO powders were identified by X-ray photoelectron spectroscopy (XPS, PHI 5000 Versa Probe, ULVAC-PHI, Kanagawa, Japan), where the Al Kα X-ray source was used at room temperature. The high energy resolution XPS spectra of La 3d, Fe 2p, Re 4f, and O 1s core levels were acquired and their binding energies (BEs) were calibration by the C1s core level line with BE values of 284.60 eV.

In order to measure the dielectric properties of the LFRO ceramic pellets, silver paste was coated at two main sides of the LFRO ceramic pellets and heated at 560 °C for 2 h to cure the electrodes. The dielectric data were measured by using a computer-controlled Agilent 4192 A impedance analyzer (Agilent technologies, Santa Clara, CA, USA) in the frequency range of 10^3^–10^6^ Hz and temperature was controlled from 30 °C to 500 °C by an automated temperature controller (DMS-2000, Partulab Technology, Wuhan, China). Magnetic properties of the LFRO powders were characterized by superconducting quantum interference device magnetometer (SQUID, Quantum Design MPMS3, San Diego, CA, USA). DC magnetizations were measured in the temperature range of 300–900 K at the field cooling (FC) and zero-field cooling (ZFC) modes, respectively. In FC mode, the applied magnetic fields were 0.1 T and 1.0 T, respectively. Optical diffuse-reflectance spectra of the LFRO powders were collected by using a spectrometer (Shimadzu UV-3150, Nakagyo-ku, Kyoto, Japan) at room temperature, and the wavelength varied from 300 nm to 800 nm. The band gap of the LFRO powder was deduced from the optical diffuse-reflectance spectra based on the Kubelka–Munk function [40]

*α*/*S* = (1 − R)^2^/2R (1)

where R stands for the reflectivity, *α* denotes the absorption coefficient, and *S* is the scattering coefficient. The photoluminescence (PL) spectrum of the LFRO powders was collected by using a Jobin Yvon spectrophotometer at 10 K, where a 325 nm laser was used as an excitation source.

## 3. Results

### 3.1. Structural Characterization 

**Figure 1:**
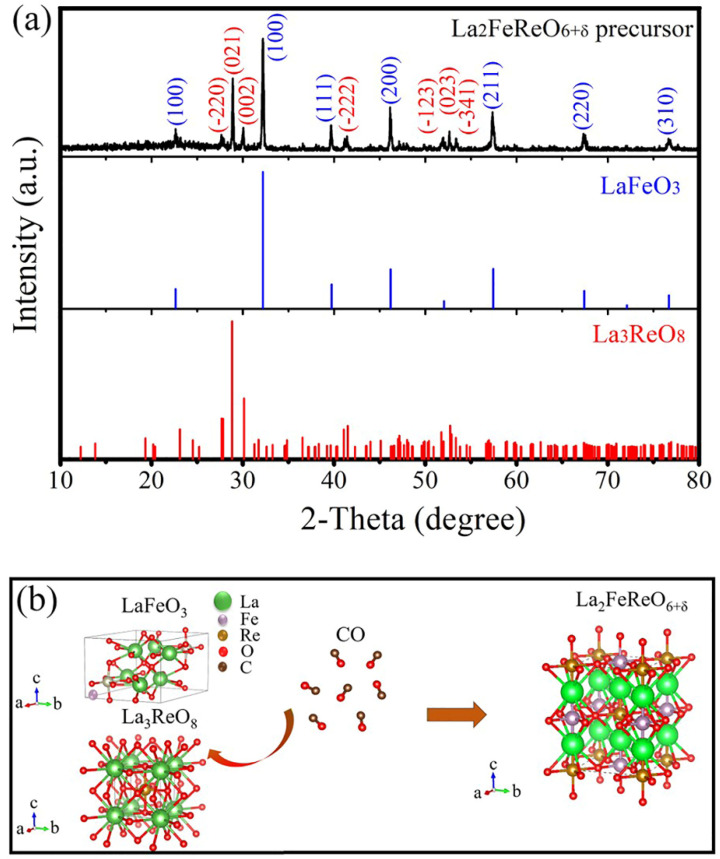
(**a**) XRD pattern of the obtained LFRO precursor. For comparison, XRD patterns of LaFeO_3_ and La_3_ReO_8_ are also presented. (**b**) Schematic diagram illustrating the reaction process of LFRO from the precursors of LaFeO_3_ and La_3_ReO_8_.

XRD pattern of the obtained LFRO precursor is shown in Figure 1a. It is observed that the LFRO precursor is composed of LaFeO_3_ (PDF card number 37-1493) and La_3_ReO_8_ (PDF card number 27-1181). The diffraction peaks contributed from LaFeO_3_ (labeled with blue color) are indexed as (100), (110), (111), (200), (211), (220), and (310), respectively, which matches well with the XRD data of perovskite LaFeO_3_ reported by Kakimoto et al. [41]. Similarly, the diffraction peaks (labeled with red color) contributed from La_3_ReO_8_ can be indexed of (220), (021), and (002), which is constituent with the XRD pattern of La_3_ReO_8_ reported by Rae-Smith et al. [42]. The valence of Re element in La_3_ReO_8_ is +7, whereas the acquisition of LFRO powders requires the reduction of Re to +4 oxide state. Figure 1b schematically shows the reaction mechanism of carbon monoxide reducing the precursors of La_3_ReO_8_ and LaFeO_3_ to La_2_FeReO_6+__δ_ powders. 

**Figure 2:**
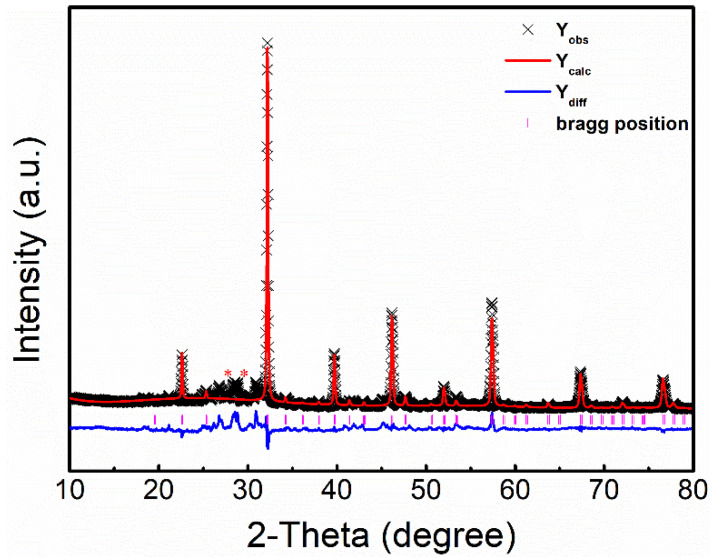
Comparison between the experimental XRD pattern of the LFRO powders and the calculated one, and their difference. The experimental and calculated data, and the difference are represented by black crosses, continuous red lines, and blue lines, respectively.

Figure 2 shows the comparison between the experimental XRD pattern of the LFRO powders and calculated ones, and their differences. Good match between the calculated and experimental XRD patterns are found, which is also confirmed by the fitting parameters R_p_ = 11.2% and R_wp_ = 15.07%. The refined lattice parameters of the LFRO powders were *a* = 5.5796 Å, *b* = 5.5896 Å, *c* = 7.8882 Å, *α* = *β* = *γ* = 90°, and the LFRO powders crystallized in an orthorhombic crystal structure with a space group of *Pbnm*. It is also noticed that some additional weak diffraction peaks (marked with red stars) appear, coming from the minor impure phase of ReO_3_. The average particle size of LFRO powders evaluated by the Scherrer equation was about 50 nm based on the (100) diffraction peak. 

Surface morphology of the as-synthesized LFRO powders revealed by the SEM image is as shown in Figure S1a, which reveals that the LFRO powders exhibit column-like morphology and a tendency of agglomeration due to the mutual attraction between the neighboring powders. The size histograms (length and width) of the column-like particles are shown in Figure S1b and Figure S1c, respectively. It was found that the length of the column-like particles was in the range of 60–400 nm, and the average length was 175 nm. The width of the column-like particles was in the range of 60–270 nm, and the average width was 110 nm. Therefore, the geometrical (length and width) dimensions of the column-like particles were larger than those estimated by the Scherrer equation. The average particle size determined from the Scherrer equation only reflects the average crystalline size perpendicular to the reflecting crystal plane (100), whereas the average particle size determined from SEM image has taken into account the wide particle size distributions of the sample. Therefore, the average particle size obtained from SEM images is much more reliable. Figure S1d presents the typical EDS spectrum collected from the LFRO powders in a mapping mode, which demonstrates the compositional signals of La, Fe, Re, and O elements. The quantitative EDS data gave out the molar ratios of La, Fe, and Re elements equal to 1.95:1.05:1.01, very close to the nominal value of 2:1:1.

### 3.2. XPS Spectra Analysis 

**Figure 3:**
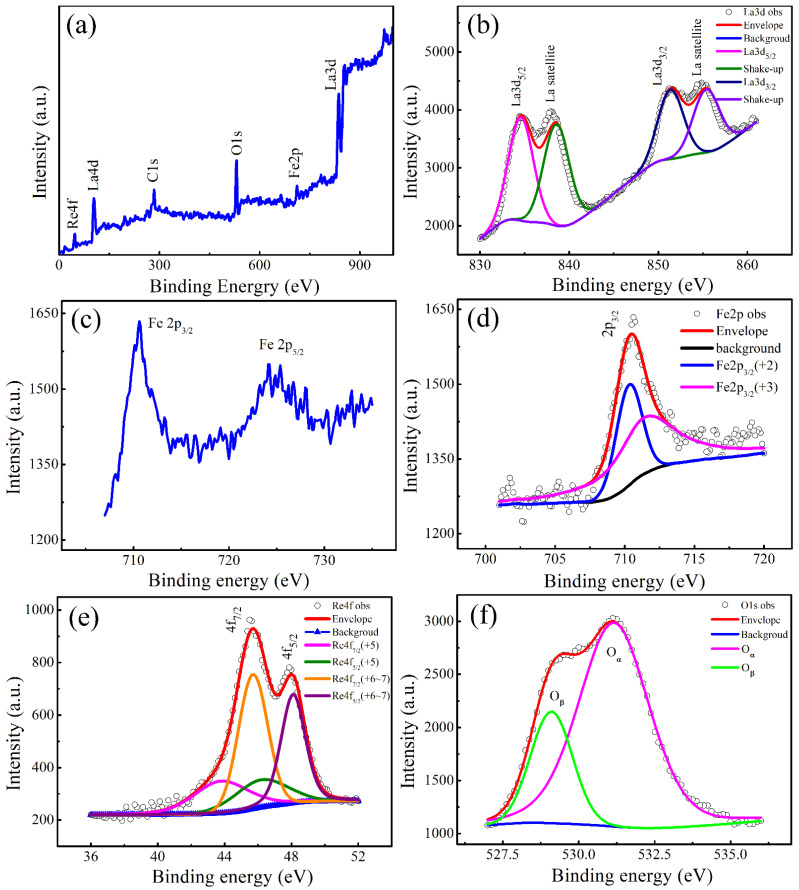
(**a**) Whole XPS spectrum of the as-synthesized LFRO powders; (**b**) local scan and peak fittings of La 3d XPS spectrum; (**c**) Fe 2p XPS spectrum and (**d**) the fitted Fe 2p_3/2_ XPS spectrum; (**e**,**f**) local scans and peak fittings of Re 4f and O 1s XPS spectra, respectively.

XPS spectroscopy is used to identify the oxidation states of the elements in the LFRO powder. Figure 3a shows the wide scanning XPS spectrum, which indicates the existence of all expected elements, namely La, Fe, Re, and O. The observed C1s XPS core level line was attributed to the LFRO powders attached to the adhesive carbon tape during XPS measurement, and its BE value (284.60 eV) was used to calibrate the BE values of La, Fe, Re, and O elements. The region scan of La 3d XPS spectrum shown in Figure 3b consists of two doublets, and their XPS peaks appear with BE positions of 834.45 eV and 851.30 eV, and 838.05 eV and 854.93 eV, respectively. The first two peaks are assigned to La 3d_5/2_ and La 3d_3/2_, respectively, indicating the existence of La^3+^ ion. The other two peaks located at 838.05 eV and 854.93 eV, are named as the shake-up satellite peaks of La 3d_5/2_ and La 3d_3/2_, respectively. Normally, as the La atom is under excitation and the photoelectron is ejected, a core hole position is left. Due to the electrons in the La atom exhibiting relaxation, the left core hole can participate in the secondary process where a valence electron from oxygen is stimulated to the empty conduction states localized in the La atom [43]. Therefore, extra energy is needed to force a valence electron from the oxygen to transfer to the core hole position in the La atom, making the photoelectron emerge at a higher BE value in the spectrum as compared with the core line. That is the reason why the shake-up satellite peaks appear at the higher energy side of the La 3d_5/2_ and La 3d_3/2_ core lines, respectively, as observed in Figure 3b. Since the intensity of the shake-up peaks represents the ability of the 2p orbital of oxygen donating lanthanum electrons, the comparable intensities between the shake-up satellite peaks and the main XPS core lines (La 3d_5/2_ and La 3d_3/2_) confirm the strong covalent nature of the La-O bond. Figure 3c is the XPS spectrum of Fe 2p, which consists of two peaks with BE positions of 724.80 eV (Fe 2p_1/2_) and 710.50 eV (Fe 2p_3/2_), respectively. These BE values match well with the XPS data of the Fe 2p for Fe_3_O_4_ samples reported by Yamashita et al. [44], indicating the presence of Fe^3+^ and Fe^2+^ ions in the LFRO powders. The absence of the satellite peak of the Fe 2p_3/2_ XPS peak is also confirmed in Figure 3c, which is in agreement with the previous report that the Fe 2p_3/2_ XPS peak in Fe_3_O_4_ does not have a satellite peak XPS peak [45]. To determine the molar ratio of [Fe^2+^] to [Fe^3+^], Fe 2p_3/2_ XPS peak was deconvoluted into Fe^2+^ and Fe^3+^ peaks with BE positions of 710.3 eV and 711.4 eV, respectively, as shown in Figure 3d. The area ratio of the two constituent peaks assigned to Fe^2+^ and Fe^3+^ gave out [Fe^2+^]:[Fe^3+^] = 0.33:0.67. Therefore, the average oxide state of the Fe ions is +2.67. The appearance of Fe^2+^ ions is ascribed to the reduction of Fe^3+^ ions under high temperature and CO reducing atmosphere and the formation of oxygen vacancies ($V_{0}^{\cdot \cdot }$) in the LFRO powders during the sample preparation. Conduction electrons are released accompanying the following ionization reaction:

(2) $$0_{0}^{\times }\to \frac{1}{2}{\;O}_{2\;}{(g)+V}_{0}^{\cdot \cdot }+{2e}^{\prime }$$

The conduction electrons can be captured by Fe^3+^ ions, described by the following equation

(3) $${Fe}^{3+}+e^{\prime }↔{Fe}^{2+}$$

Therefore, Fe^3+^ ions are reduced to Fe^2+^ ions, resulting in the dual chemical valence states (Fe^2+^ and Fe^3+^ ions) in the LFRO powders. Figure 3e shows the local scan Re 4f XPS spectrum, which displays the characteristics of 4f doublet separated by 2.3 eV (Re 4*f*_7/2_ and 4*f*_5/2_ with BE positions of 45.6 eV and 47.9 eV, respectively) due to the spin-orbit splitting. In order to fit the Re 4*f* peaks two doublets with BE positions of 43.8 eV and 46.2 eV, and of 45.7eV and 48.1 eV, are required. The former two components are assigned to Re^5+^ species [46], while the latter two ones can be assigned to Re^6−7+^ species because its Re 4f_7/2_ BE value (45.7 eV) is 0.8 eV larger than that reported for ReO_3_ (Re^6+^, BE = 44.9 eV) [47] but 1.1 eV smaller than that for Re_2_O_7_ (Re^7+^, BE = 46.8 eV) [48]. Based on the areas under these peaks, the concentration ratio of [Re^5+^] to [Re^6−7+^] species was determined to be 0.33:0.67. However, by assuming a contribution of the three oxidation states (Re^5+^, Re^6+^, and Re^7+^) to the Re 4f XPS spectrum, and the contributions of Re^5+^, Re^6+^, and Re^7+^ ions are found to be 33%, 39%, and 28%, respectively. Thus, the average oxide state of the Re ions is +5.95. Figure 3f displays the local XPS spectrum of O 1s, where an asymmetric peak is observed and it is deconvoluted into two sub-peaks, indicating the presence of two types of oxygen species. The sub-peak with higher BE value (531.16 eV) is assigned to the adsorbed oxygen species (denoted as O_α_) in this kind complex oxide with oxygen deficiency [49], while the other one with lower BE value (529.18 eV) corresponds to the lattice oxygen (O^2−^) in the crystal structure of LFRO (denoted as O_β_) [50]. Based on a series of O1s XPS data of metal oxides, hydroxides, and peroxides, Dupin et al. [51] assigned the O 1s XPS peaks with BE values in the range of 531–532 eV to the “O^−^” ions. These ionizations of oxygen species are associated with sites where the coordination numbers of oxygen ions are smaller than those in regular sites, exhibiting a higher covalence of the M-O bonds and allowing for the compensation of deficiencies in the subsurface of transition metal oxides. Due to a higher covalence of the M-O bonds these low coordinated oxygen ions have a lower electron density than the classical “O^2−^” ions. It is noticed that the concentration of the adsorbed oxygen is higher, which implies more absorbed oxygen in this kind complex oxide at the surface. To maintain the charge balance within the unit cell of the LFRO crystal (the total positive charges contributed from La, Fe, and Re cations are +14.26), so the required oxygen species should include six lattice oxygen (O^2−^) and about 3 “O^−^” ions. That is the reason why more absorbed oxygen species (“O^−^” ions) are found at the surface of this kind complex oxide, which contribute to the component of O 1s XPS spectrum with BE positons in the range of 531–532 eV. 

### 3.3. Dielectric Properties

**Figure 4:**
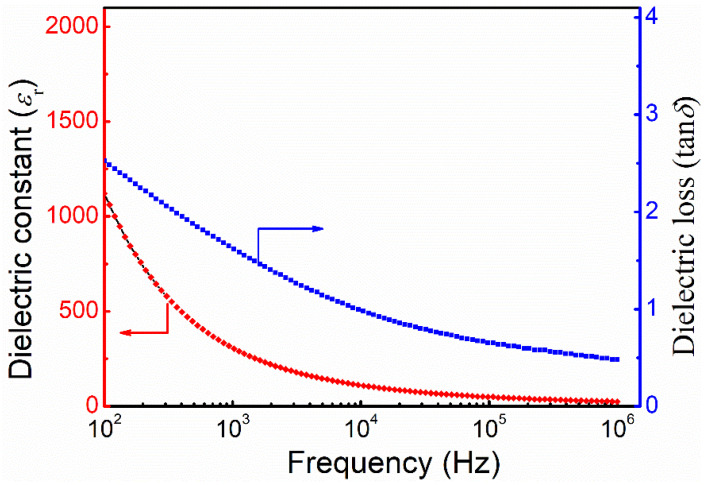
Dielectric constant (*ε*_r_) and dielectric loss (tan *δ*) of the LFRO ceramic sample measured at room temperature as a function of frequency.

Figure 4 presents the variations of dielectric constant (*ε*_r_) and dielectric loss (tan*δ*) of the LFRO ceramic pellets with frequency at room temperature. It is found that both the dielectric parameters exhibit decreasing trend with increasing the measured frequency. That is a fast reduction in the low-frequency region and a tendency towards stabilization in the high-frequency region. The LFRO ceramics has much large values of *ε*_r_ and tan*δ* at low frequency (e.g., *ε*_r_ = 1125 and tan*δ* = 2.5 @ 100 Hz). This dielectric dispersion at low frequency can be ascribed to a comprehensive reactions of the $V_{0}^{\cdot \cdot }$ induced dielectric relaxation and Maxwell–Wagner type of interfacial polarization, as well as the HM metallic character of this compound. The temperature dependence of the dielectric properties of the LFRO ceramics were measured at several selected frequencies, as depicted in Figure S2. Figure S2a is the variation of *ε*_r_ as a function of the temperature from 30 °C to 500 °C, where the *ε*_r_ increases slowly from 30 °C to 310 °C, and then increases rapidly as the temperature is beyond 350 °C. The *ε*_r_ achieves a maximum value around 450 °C and then rapidly decreases. It was also noticed that the temperature (*T*_m_) at which the *ε*_r_ achieved a maximum value shifted towards the higher temperature with increasing the frequency from 1 kHz to 1 MHz. Furthermore, the maximum value of *ε*_r_ reduces as the frequency increases. That suggests the LFRO ceramics displays a relaxor-like dielectric relaxation phenomenon in the temperature range of 300–500 °C, which is ascribed to the disorder in the crystalline structure of the LFRO ceramics. It is reported that the nature of the structural disorder is different for different groups of relaxors [52]. Generally, relaxors can be classified as a structural disorder accompanied by variations of the local electric fields such as the case of PbMg_1/3_Nb_2/3_O_3_ (with polar nanodomains), or the local strain fields such as the case of (Pb,Ba)(Zr,Ti)O_3_, and a structural disorder accompanied by $V_{0}^{\cdot \cdot }$ such as the case of ((Pb_1−3x/2_La_x_)(Zr_0.4_Ti_0.6_)O_3_:PLZT) [52]. In the present complex double perovskite LFRO compound, there exists $V_{0}^{\cdot \cdot }$ as described by Equation (2) and the different oxidation states of the Fe (Fe^2+^ and Fe^3+^) and Re (Re^5+^, Re^6+^ and Re^7+^) cations at B-site, as revealed by XPS spectra, which provide the hopping paths for the electrons (e.g., between Fe^2+^ and Fe^3+^ ions, or Re^5+^ and Re^6+^ or Re^7+^ ions). The structural disorder nature at B-site in LFRO ceramics will lead to a distribution of relaxation times. The heights of the energy barriers for electron hopping from one site to another are also not identical for all the sites in the LFRO ceramics. The hopping of electrons among the heterovalent cations at B-site, and the oxygen ion hopping through the vacant oxygen sites will lead to the rotation of dipoles, which result in the relaxor dielectric behavior at temperatures around 450 °C, as observed in Figure S2a.

Figure S2b illustrates the tan*δ* as a function of the temperature from 30 °C to 500 °C. The tan*δ* increases slowly as the temperature below 150 °C, and then increases fast from 200 °C to 400 °C. A sharp increase is observed at temperature beyond 450 °C, which can be ascribed to the thermal activation of $V_{0}^{\cdot \cdot }$ in the ceramic samples [53]. The localized electron hopping between the heterovalent cations (e.g., Fe^2+^ and Fe^3+^, or Re^5+^ and Re^6+^ or Re^7+^ ions) at B-site under a reducing CO atmosphere also increases the conductivity, especially in the present HM metallic LFRO compound, leading to the fast increase of tan*δ* at high temperature. At the temperature below 150 °C, the values of*ε*_r_ and tan*δ* are much small, which is ascribed to the fact that the $V_{0}^{\cdot \cdot }$ generated during high-temperature sintering process are difficult to move freely because of the strong combination with LFRO crystal lattice defects [29]. 

In order to reveal the nature of dielectric relaxation behavior of the LFRO samples, the dielectric data were fitted by a nonlinear Vogel−Fulcher law as

(4) $$\omega =\omega_{0}exp\left(\frac{-E_{a}}{k_{B}(T_{m}-T_{f})}\right)$$

where *ω* is the angular frequency, *ω*_0_ is the pre-exponential factor, *k*_B_ is Boltzmann constant, *T*_f_ is the freezing temperature at which the dipolar cluster polarization is no longer thermally activated, *E*_a_ is activation energy, and *T*_m_ is temperature where the maximum value of *ε*_r_ achieves at the angular frequency *ω*. Based on the plot of *ω* vs. *T*_m_ shown in Figure S2c, the *E*_a_ value (0.05 eV) of the dielectric relaxation process can be deduced from the best fitting procedure of the Vogel−Fulcher law to the dielectric data. This *E*_a_ value matches well with the dissociation energy (0.05–0.06 eV) required to excite an electron from the donor neighborhood into an unbound small-polaron state in n-type BaTiO_3_ ceramics [54]. That indicates the jumping of electrons trapped in the shallower level created by oxygen vacancy also contributes to the relaxor-like dielectric behavior observed at temperatures around 450 °C, as observed in Figure S2a. 

### 3.4. Magnetic Properties 

**Figure 5:**
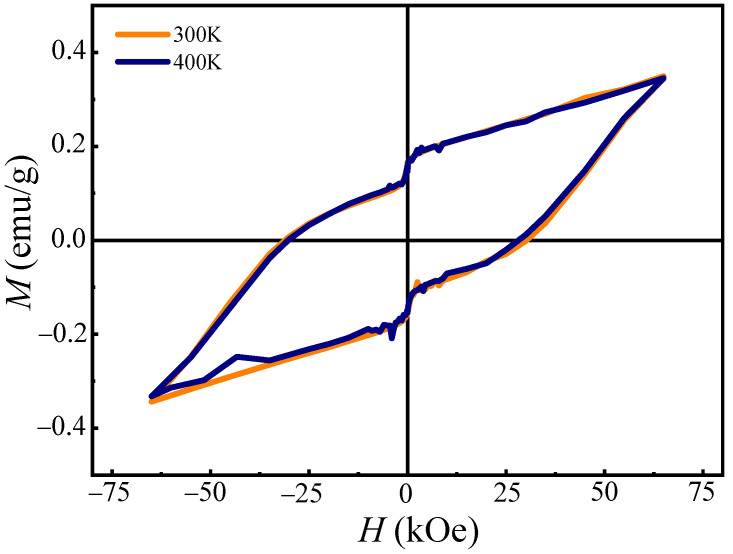
*M-H* hysteresis loops of the LFRO powders measured at 300 and 400 K, respectively.

The *M-H* hysteresis loops of the LFRO powders measured at 300 K and 400 K, respectively are shown in Figure 5. It is interesting that the two *M-H* hysteresis loops exhibit almost the same curves despite of the different measured temperature. The residual magnetization and coercive field were measured to be 0.160 emu/g and 28.85 kOe, respectively. Furthermore, they did not achieve the saturation state in spite of a magnetic field as high as 70 kOe, indicating that there was an AFM interaction in the LFRO samples. Figure S3a displays the dc magnetizations of the LFRO powders as a function the temperature measured under ZFC and FC modes and different applied fields (e.g., 1 kOe and 10 kOe). From which the magnetic transition from PM to FM phases was determined to be 746 K. A bifurcation between the magnetizations measured under ZFC and FC modes and magnetic field of 1 kOe, was observed 758 K, and it increased with the decrease of temperature. Similar case was also observed under magnetic field of 10 kOe. This phenomenon usually appears in the systems that have both FM and AFM magnetic moments, which is weaker under 1 kOe magnetic field [55]. Small peaks at 748 K and 737 K, were observed in the ZFC magnetization curves under the magnetic field of 1 kOe and 10 kOe, respectively. 

The appearance of this peak is generally considered to be resulting from the superparamagnetic or spin glass behavior of the sample [56]. As demonstrated in Figure S3a, below the peak temperature, the *M*_FC_ increases slowly with decreasing the temperature. Therefore, the peaks observed that the *M*_ZFC_-*T* curves originate from the presence of superparamagnetic states in the LFRO powders. Figure S3b demonstrates inverse dc magnetic susceptibilities (*χ*^−1^) of the FC curves measured under magnetic fields of 1 kOe and 10 kOe in high temperature region, which are well fitted by Curie–Weiss law. The Curie–Weiss constant (*C*) and temperature (*θ*_p_) were extracted from the fitting, which were 1.69 K·emu/(mol·Oe) and 626 K (@1 kOe), and 1.76 K·emu/(mol·Oe) and 385 K (@ 10 kOe), respectively. Strong FM interactions are inferred from the large and positive *θ*_p_ values in the LFRO powders, and the effective paramagnetic moment (*μ*_eff_) can be evaluated by the fitted Curie constant *C* via the following equation [57]:

(5) $$\mu_{\mathrm{eff}}=\sqrt{\frac{3k_{B}C}{N_{A}\mu_{B}^{2}}}=2.827\sqrt{C}$$

where *N*_A_, *μ*_B_ and *k*_B_ have the normal meanings as in the textbook. Thus, the *μ*_eff_ values were estimated to be 3.67 *μ*_B_ (@ 1 kOe) and 3.75 *μ*_B_ (@ 10 kOe), respectively. By assumption of only a pair of Fe and Re ions in a unit cell of the LFRO compound (an insulator), the theoretical magnetic moment *μ*_cal_ per formula unit for the La_2_(${\mathrm{Fe}}_{0.33}^{2+}{\mathrm{Fe}}_{0.67}^{3+}$)(Re0.335+Re0.396+Re0.287+)O_6+δ_ compound can be calculated by Equation (6) [58]

(6) μcal=0.33×(μFe2+)2+0.67×(μFe3+)2+0.33×(μRe5+)2+0.39×(μRe6+)2  =5.76 μB

where the data of $\mu_{{\mathrm{Fe}}^{2+}}=4.899\;\mu_{B}$, $\mu_{{\mathrm{Fe}}^{3+}}=5.916\;\mu_{B}$, μRe5+=1.633 μB, μRe6+=1.549 μB,  are used [59]. The *μ*_eff_ value is much smaller than the theoretical *μ*_cal_, which is due to the presence of AFM interactions between Fe^2+^/Fe^3+^ and Re^5+^/Re^6+^ (or Re^7+^) ions in the LFRO powders. In the χ^−1^-*T* curve, there exists a sharp downturn deviation from the Curie–Weiss law as the temperature is reduced. That represents the characteristics of Griffith phase (GP) [60], which meets the power law

(7) $$\chi^{-1}{\left(T)\;\propto \;(T-T_{C}^{R}\right)}^{1-\lambda }\;(0\;\leq \;\lambda \;<\;1.0)\;$$

where λ is the magnetic susceptibility exponent, describing the strength of GP, and the value of $T_{C}^{R}$ can be obtained from *λ* = 0 in the Curie–Weiss regime [61], which is equivalent of the *θ*_P_. Clearly, *λ* = 0 corresponds the normal Curie–Weiss law. In the high temperature region (the PM state), *χ*^−1^ versus *T* curve exhibits a linear Curie–Weiss behavior, however, it displays a deviation from the linearity as reducing the temperature where a sharp downturn is observed at the common point corresponding to $T_{C}^{R}$. The abruptness of this downturn was found to be decreased with increasing the magnetic field, as compared the two *χ*^−1^ vs. *T* curves shown in Figure S3b, and the $T_{C}^{R}$ value was determined to be 758 K. Normally, the feature of the GP phase is described as the presence of FM-correlated finite clusters with disorder PM phase as a background. This mixed phase exists in the temperature range of *T*_C_ ≤ *T* ≤ $T_{C}^{R}$ due to disorder quenching [60]. Figure S3c and Figure S3d present the plots of ln*χ*^−1^ vs. ln (*T*−$T_{C}^{R})$ measured at 1 kOe and 10 kOe, respectively, which exhibit a linear behavior at low value of (*T*−$T_{C}^{G}).$ That confirms the presence of GP phase in the LFRO powders. The *λ*_GP_ values at 1 kOe and 10 kOe were extracted from the linear fittings, which were 0.978 and 0.992, respectively. These values are comparable with those reported for the La_1−x_Ca_x_Mn_0.9_Cu_0.1_O_3_ samples with *λ*_GP_ = 0.983 (x = 0.3) and *λ*_GP_ = 0.973 (x = 0.4) [62]. 

### 3.5. Optical Properties 

**Figure 6:**
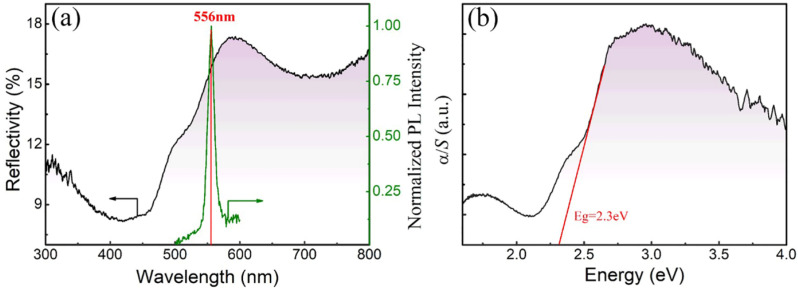
(**a**) Optical diffuse reflectance spectrum of the LFRO powders. Inset is a steady-state photoluminescence (PL) spectrum measured at 10 K under laser beam excitation with wavelength of 325 nm. (**b**) Plot of *α*/S vs. *hν* curve derived from the Kubelka–Munk function.

For obtaining the band gap of the LFRO powders, optical diffuse-reflectance spectrum was measured. Figure 6a shows the reflectivity spectrum of the LFRO powders measured from 300 nm to 800 nm. It is observed that the reflectivity rises sharply near 500 nm, which indicates that the density of shallow-level defects is low and the sample is relatively pure. Kubelka–Munk function is utilized to estimate the band gap in LFRO powders. The plot of *α/S* versus *hν* curve is shown in Figure 6b, which exhibits a linear relationship near the absorption edge. The optical band gap (*E*_g_ = 2.30 eV) can be obtained by extrapolating the tangent linear to the intercept of the *x*-axis (*hν*), The obtained *E*_g_ value is 2.30 eV, close to the direct band gap (*E*_g_ = 2.34 eV) of epitaxial LaFeO_3_ films [63]. Such direct band gap is contributed from the electronic transition from O 2p to Fe 3d orbitals. Furthermore, a steady-state PL spectrum of the as-synthesized LFRO powders was measured under laser excitation with λ = 325 nm at 10 K, to investigate their charge-carrier recombination process. An inset in Figure 6a is a sharp green PL peak at 556 nm with a FWHM (full width at half maximum) of 13 nm, which reveals the photo-induced electrons and holes recombining radiatively in the LFRO due to the laser excitation. From the PL spectrum the *E*_g_ value of the LFRO powders is estimated to be 2.23 eV, close to that obtained from the optical diffuse-reflectance spectrum. The LFRO powders with *E*_g_ = 2.30 eV can effectively absorb the visible light of the solar spectrum, making them useful for photocatalytic and related solar applications. 

## 4. Conclusions

In summary, structural, dielectric, magnetic, and optical properties of the La_2_FeReO_6+__δ_ powders synthesized by solid-state reaction under reducing atmosphere of CO, are investigated. Structural Reitveld refinements on the XRD data demonstrate that the LFRO powders have an orthorhombic crystal structure with *Pbnm* space group. SEM images demonstrated that the LFRO powders exhibited column-like morphology with slight agglomeration and the particle sizes were in the range of 40–450 nm. XPS spectra verified the presence of La^3+^ ions in the LFRO powders and the mixed chemical states of Fe ions with [Fe^2+^]:[Fe^3+^] = 0.33:0.67 and Re ions with [Re^5+^]:[Re^6+^]:[Re^7+^] = 0.33:0.39:0.28. O 1s XPS spectrum revealed two kinds of oxygen species (lattice oxygen and absorbed oxygen). The LFRO ceramics display a relaxor-like dielectric relaxation behavior, which is well described by nonlinear Vogel−Fulcher law. The observed relaxation dielectric behavior could be ascribed to the hopping of electrons among the hetero-valence ions at B-site, and oxygen ions hopping through the vacant oxygen sites in the LFRO ceramics. The obtained activation energy of 0.05 eV, matches well with the dissociation energy required to excite an electron from the donor neighborhood into an unbound small-polaron state in n-type BaTiO_3_ ceramics, indicating that the jumping of electrons trapped in the shallower level created by oxygen vacancy may also be a cause of the relaxor-like dielectric behavior. The LFRO powders exhibit ferromagnetic behavior at room temperature, and the remanent magnetization and coercive field are 0.16 emu/g and 28.85 kOe, respectively. The magnetic Curie temperature, *T*_C_ was 746 K and the Griffiths temperature was 758 K below which the Griffiths-like phase began to appear in the LFRO powders. The LFRO powders have a direct band gap of 2.30 eV, as confirmed by the UV–visible diffuse-reflectance spectrum and the PL spectrum, which originates from the electronic transition from O 2p to Fe 3d orbitals. 

## Data Availability

The data presented in this work are available on request from the corresponding author. The data are not publicly available due to privacy.

## Supplementary Materials

The following supporting information can be downloaded at: https://www.mdpi.com/article/10.3390/nano12020244/s1, Figure S1: (**a**) FE-SEM image of the as-synthesized LFRO powders, and (**b**,**c**) the corresponding size histograms (length and width) of the column-like particles, respectively. (d) EDS spectrum of the as-synthesized LFRO powders; Figure S2: (**a**) Dielectric constant (*ε*_r_) and (**b**) dielectric loss (tan*δ*) of the LFRO ceramics measured from 250 °C to 500 °C at different frequencies. Inset is the dielectric constant measured from 50 °C to 250 °C at different frequencies. (**c**) Plot of ω vs. *T*_m_ curve of the LFRO ceramics; Figure S3: (**a**) Temperature dependent dc magnetizations of the LFRO powders obtained under ZFC and FC modes and different magnetic fields (e.g., 1 kOe and 10 kOe). (**b**) Inverse dc magnetic susceptibilities (*χ*^−1^) vs. *T* curves measured under FC mode and magnetic fields of 1 kOe and 10 kOe, respectively. The curves of *χ*^−1^ vs. *T* follow a linear Curie-Weiss behavior in the high temperature region, which are well fitted by solid direct lines. (**c**,**d**) Plots of ln*χ*^−1^ vs. ln (*T*−$T_{C}^{G})$ measured under FC mode and with magnetic fields of 1 kOe and 10 kOe, respectively. From their linear fittings the *λ*_GP_ values at 1 kOe and 10 kOe, can be determined.
