# Supplementary material for: Structural Characterization and Physical Properties of Double Perovskite La2FeReO6+δ Powders
Source: Nanomaterials (Basel). 2022 Jan 13;12(2):244. doi: 10.3390/nano12020244 (PMC8779002; doi:10.3390/nano12020244)
Supplement: Supplementary file 1 [file nanomaterials-12-00244-s001.zip › nanomaterials-1533840-supplementary.pdf]

nanomaterials

Supplemental Materials

Structural Characterization and Physical Properties of Double Perovskite $La_2FeReO_{6+\delta}$ Powders

Qingkai Tang and Xinhua Zhu *

National Laboratory of Solid State Microstructures, School of Physics, Nanjing University, Nanjing 210093, China; qktang@mail.nwpu.edu.cn
* Correspondence: xhzhu@nju.edu.cn

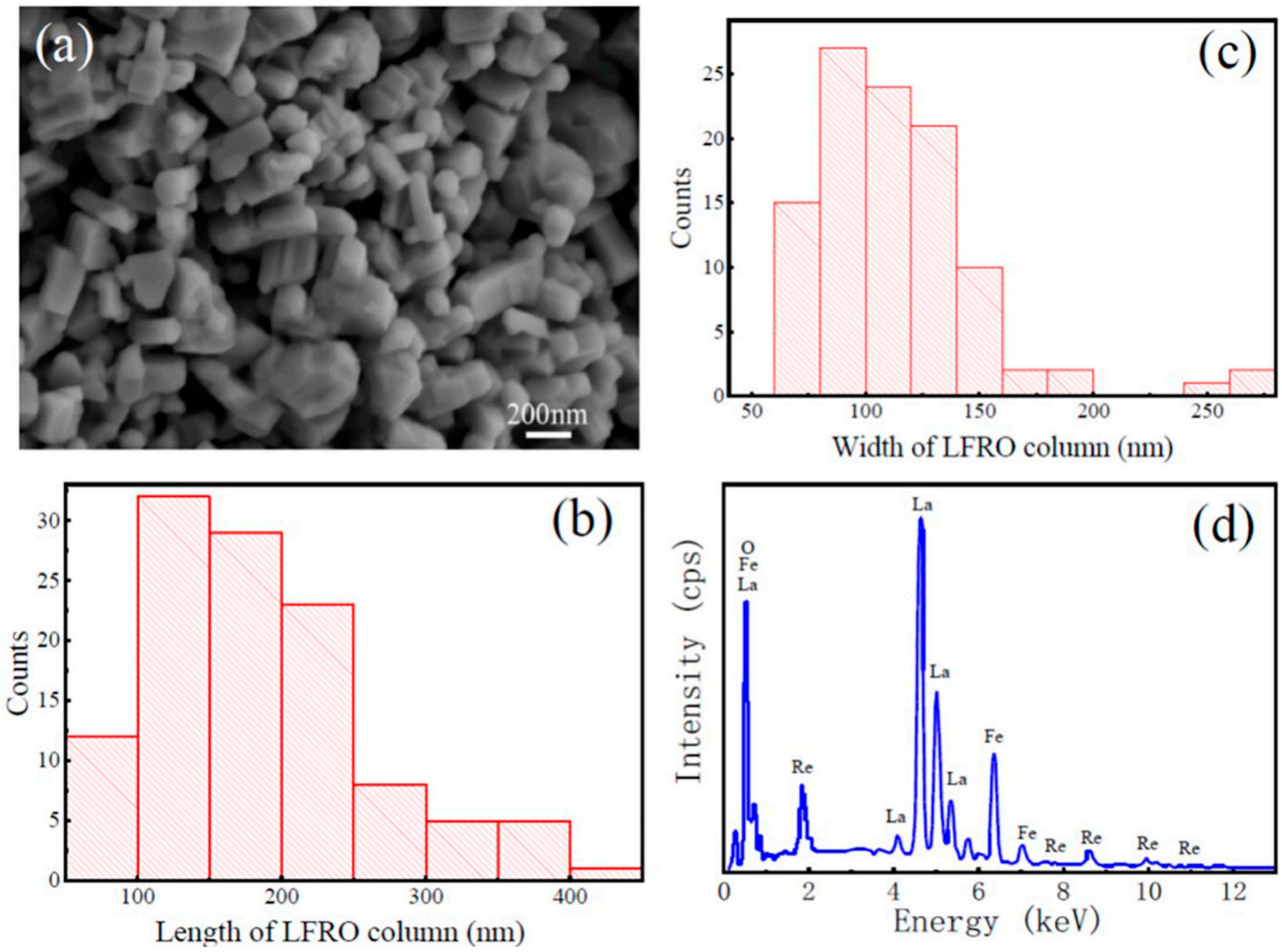

Figure S1. (**a**) FE-SEM image of the as-synthesized LFRO powders, and (**b**,**c**) the corresponding size histograms (length and width) of the column-like particles, respectively. (**d**) EDS spectrum of the as-synthesized LFRO powders.

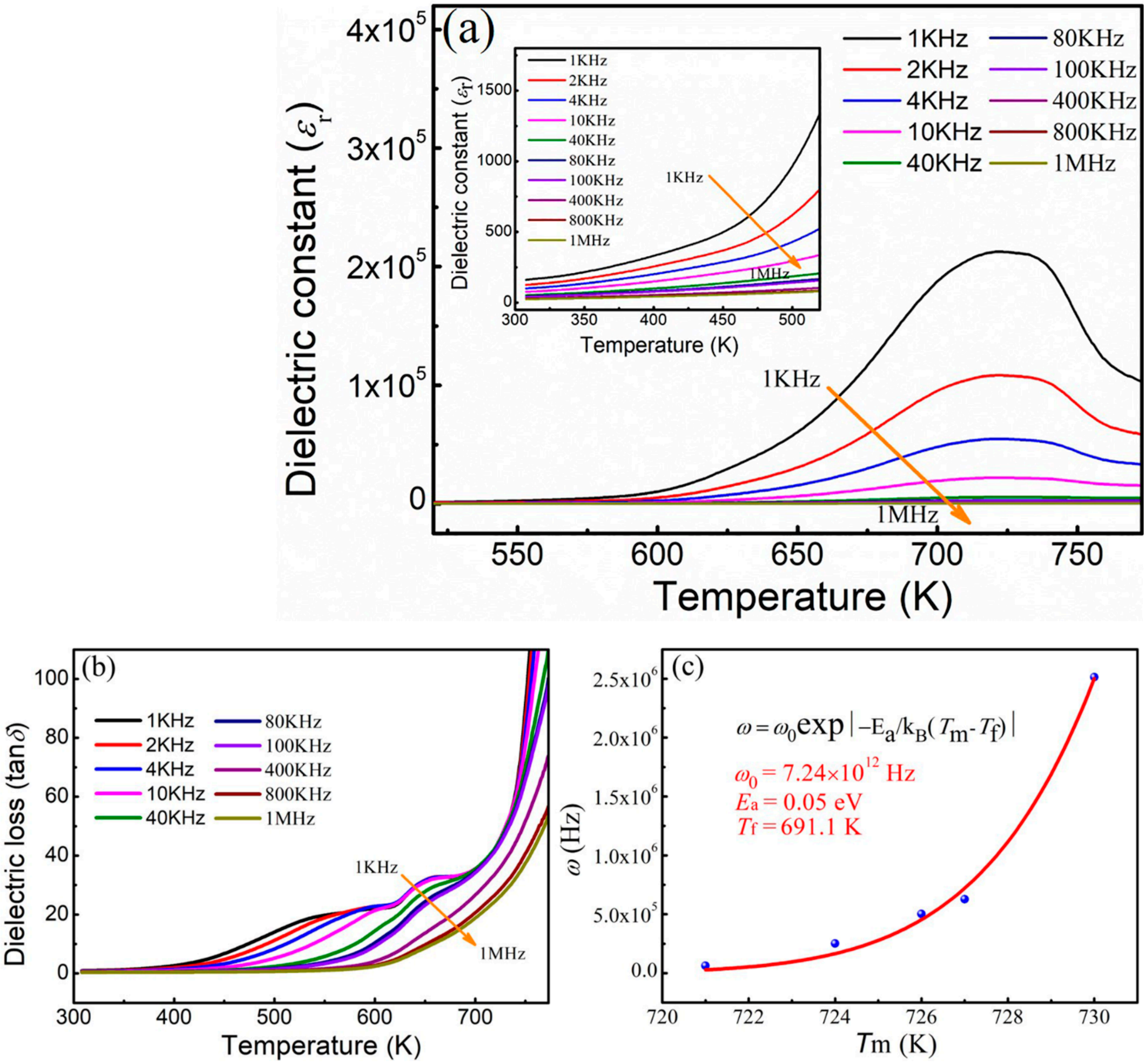

Figure S2. (**a**) Dielectric constant (ε_r) and (**b**) dielectric loss (tan δ) of the LFRO ceramics measured from 250 °C to 500 °C at different frequencies. Inset is the dielectric constant measured from 50 °C to 250 °C at different frequencies. (**c**) Plot of ω *vs.* T_m curve of the LFRO ceramics.

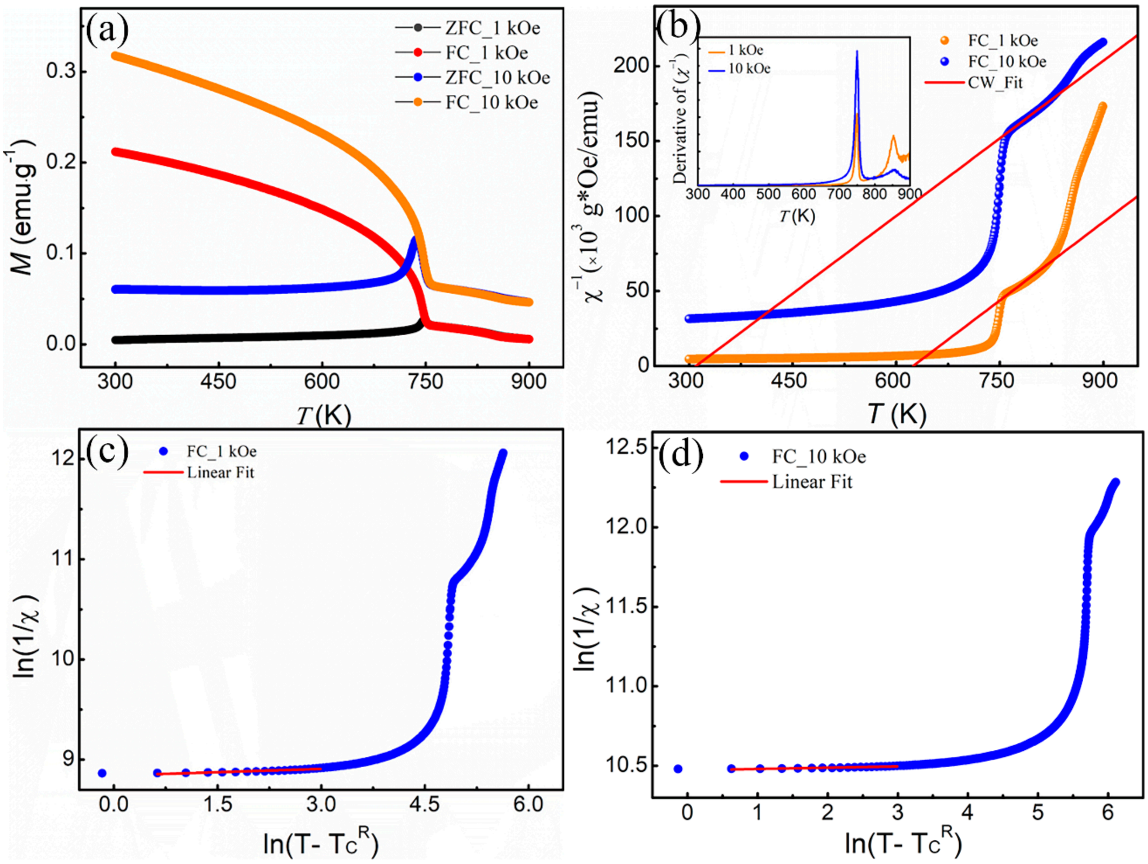

Figure S3. (**a**) Temperature dependent dc magnetizations of the LFRO powders obtained under ZFC and FC modes and different magnetic fields (e.g., 1 kOe and 10 kOe). (**b**) Inverse dc magnetic susceptibilities (χ^{1}) *vs.* T curves measured under FC mode and magnetic fields of 1 kOe and 10 kOe, respectively. The curves of χ^{1} *vs.* T follow a linear Curie-Weiss behavior in the high temperature region, which are well fitted by solid direct lines. (**c**,**d**) Plots of lnχ^{1} *vs.* ln (T-T_C^G) measured under FC mode and with magnetic fields of 1 kOe and 10 kOe, respectively. From their linear fittings the λ_{GP} values at 1 kOe and 10 kOe, can be determined.
